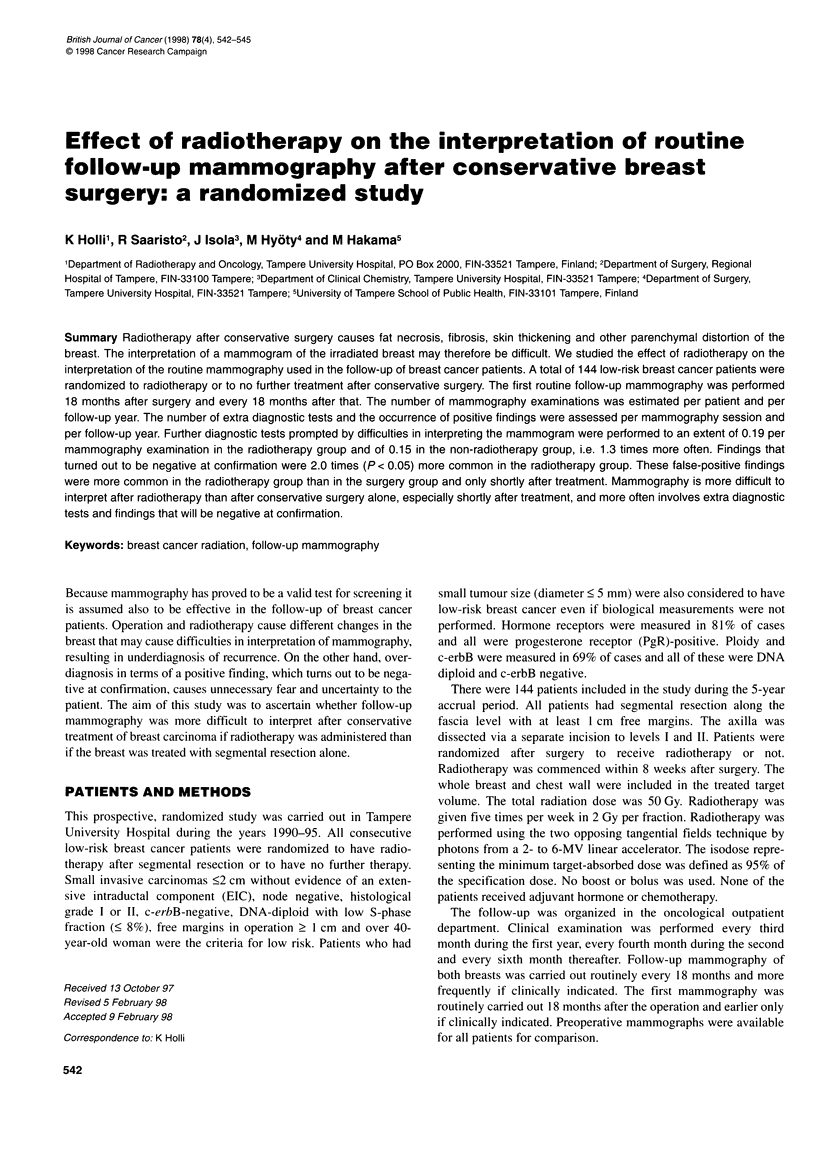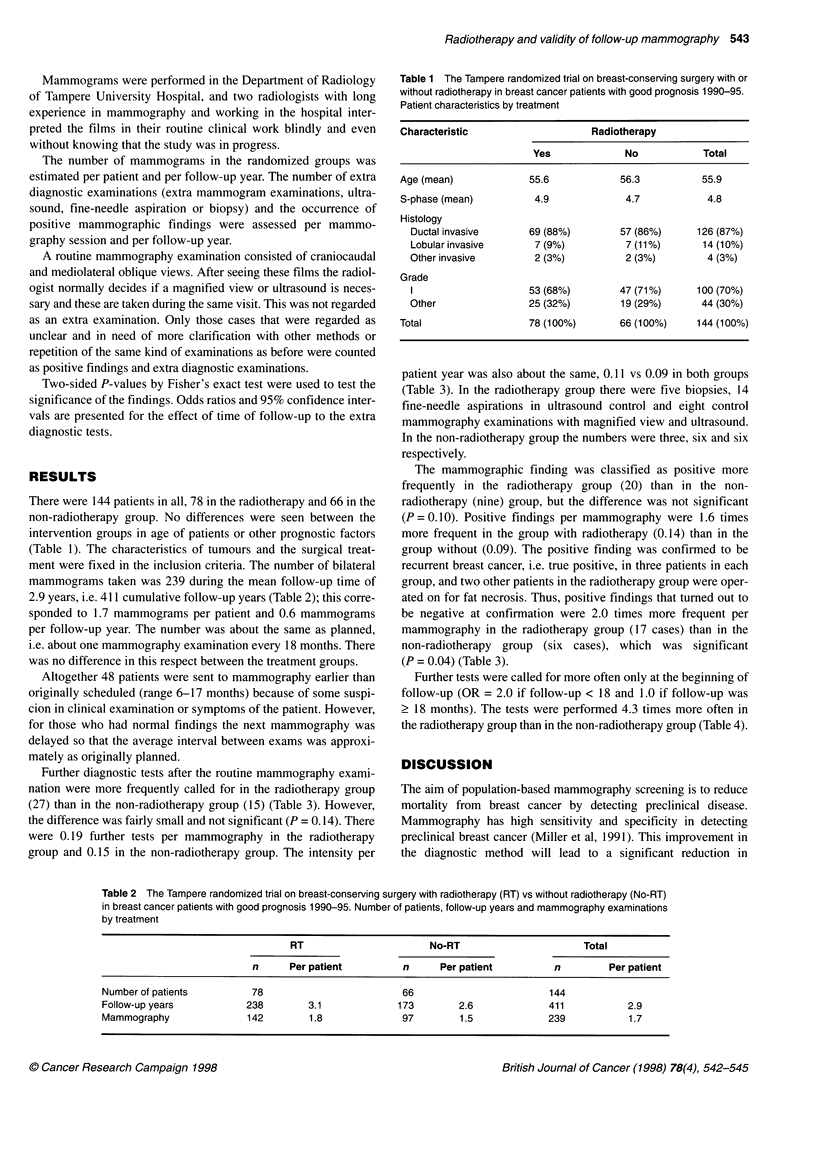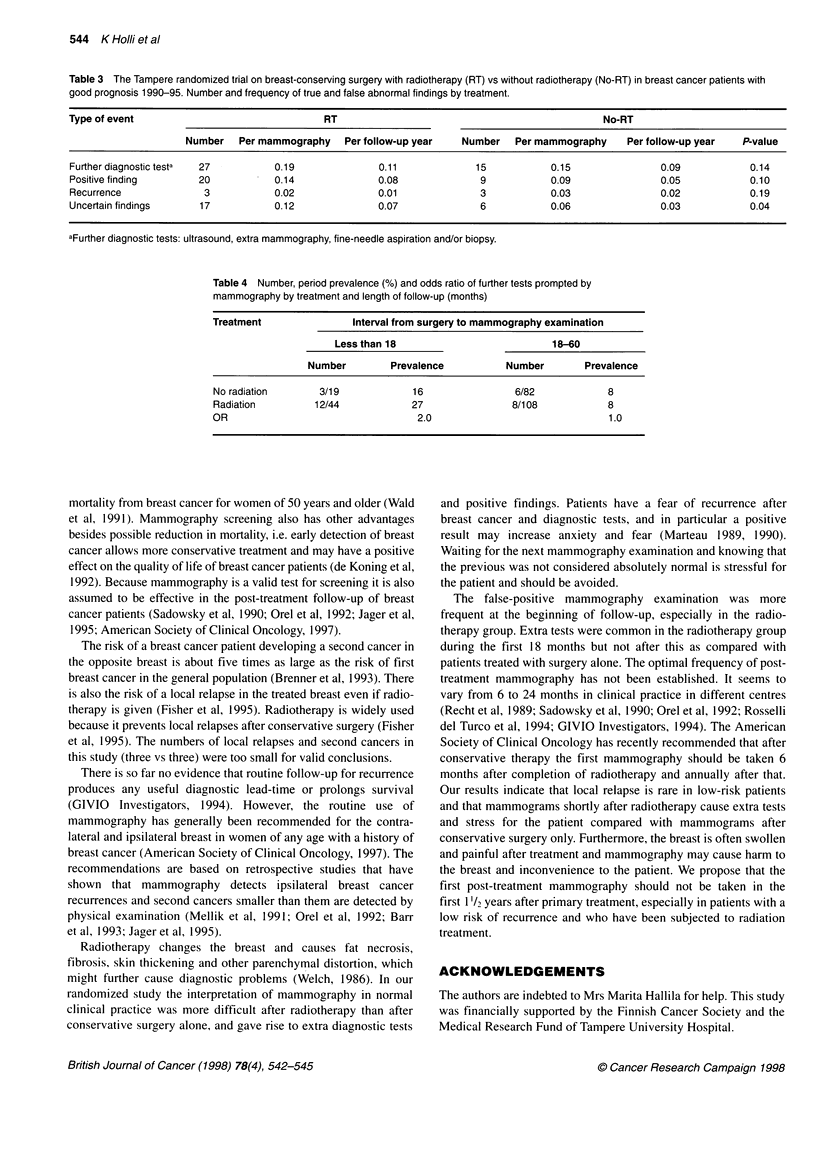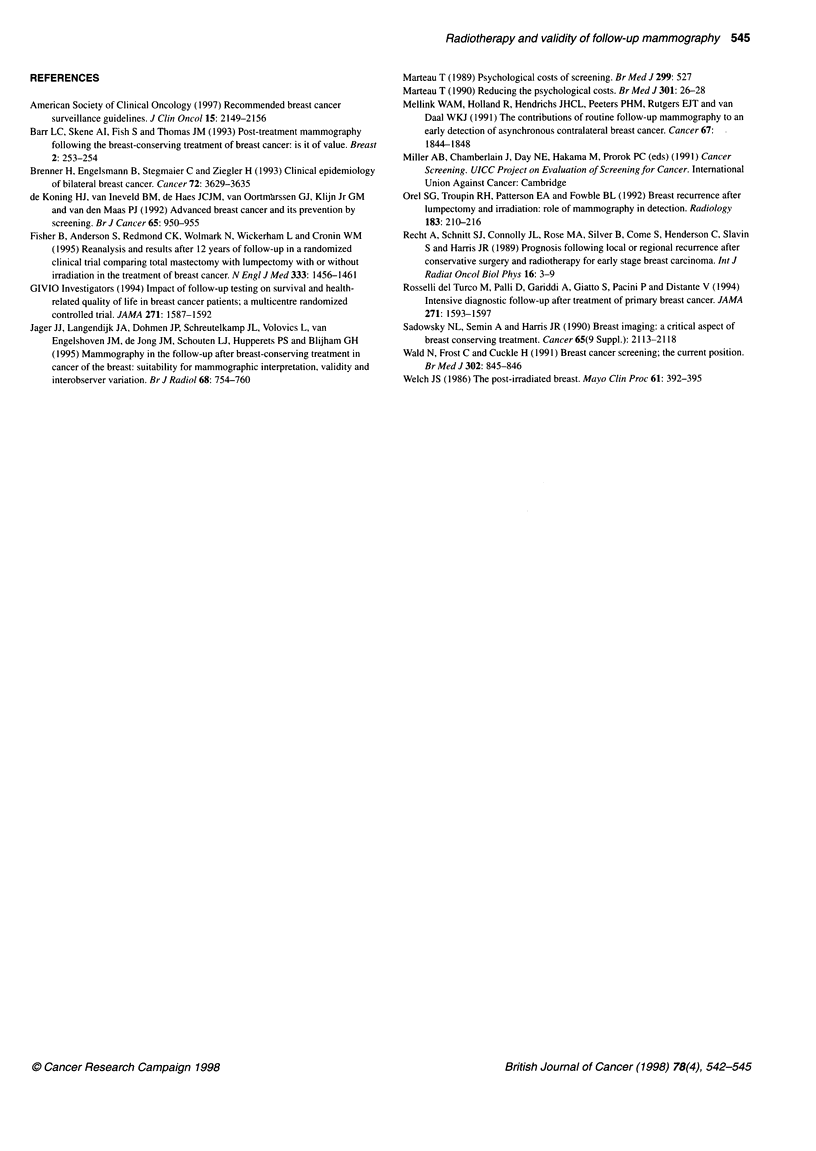# Effect of radiotherapy on the interpretation of routine follow-up mammography after conservative breast surgery: a randomized study.

**DOI:** 10.1038/bjc.1998.529

**Published:** 1998-08

**Authors:** K. Holli, R. Saaristo, J. Isola, M. Hyöty, M. Hakama

**Affiliations:** Department of Radiotherapy and Oncology, Tampere University Hospital, Finland.

## Abstract

Radiotherapy after conservative surgery causes fat necrosis, fibrosis, skin thickening and other parenchymal distortion of the breast. The interpretation of a mammogram of the irradiated breast may therefore be difficult. We studied the effect of radiotherapy on the interpretation of the routine mammography used in the follow-up of breast cancer patients. A total of 144 low-risk breast cancer patients were randomized to radiotherapy or to no further treatment after conservative surgery. The first routine follow-up mammography was performed 18 months after surgery and every 18 months after that. The number of mammography examinations was estimated per patient and per follow-up year. The number of extra diagnostic tests and the occurrence of positive findings were assessed per mammography session and per follow-up year. Further diagnostic tests prompted by difficulties in interpreting the mammogram were performed to an extent of 0.19 per mammography examination in the radiotherapy group and of 0.15 in the non-radiotherapy group, i.e. 1.3 times more often. Findings that turned out to be negative at confirmation were 2.0 times (P< 0.05) more common in the radiotherapy group. These false-positive findings were more common in the radiotherapy group than in the surgery group and only shortly after treatment. Mammography is more difficult to interpret after radiotherapy than after conservative surgery alone, especially shortly after treatment, and more often involves extra diagnostic tests and findings that will be negative at confirmation.


British Journal of Cancer (1998) 78(4), 542–545
© 1998 Cancer Research Campaign

### Effect of radiotherapy on the interpretation of routine follow-up mammography after conservative breast surgery: a randomized study

**K Holli[1], R Saaristo[2], J Isola[3], M Hyöty[4] and M Hakama[5]**

[1]Department of Radiotherapy and Oncology, Tampere University Hospital, PO Box 2000, FIN-33521 Tampere, Finland; [2]Department of Surgery, Regional Hospital of Tampere, FIN-33100 Tampere; [3]Department of Clinical Chemistry, Tampere University Hospital, FIN-33521 Tampere; [4]Department of Surgery, Tampere University Hospital, FIN-33521 Tampere; [5]University of Tampere School of Public Health, FIN-33101 Tampere, Finland

**Summary** Radiotherapy after conservative surgery causes fat necrosis, fibrosis, skin thickening and other parenchymal distortion of the breast. The interpretation of a mammogram of the irradiated breast may therefore be difficult. We studied the effect of radiotherapy on the interpretation of the routine mammography used in the follow-up of breast cancer patients. A total of 144 low-risk breast cancer patients were randomized to radiotherapy or to no further treatment after conservative surgery. The first routine follow-up mammography was performed 18 months after surgery and every 18 months after that. The number of mammography examinations was estimated per patient and per follow-up year. The number of extra diagnostic tests and the occurrence of positive findings were assessed per mammography session and per follow-up year. Further diagnostic tests prompted by difficulties in interpreting the mammogram were performed to an extent of 0.19 per mammography examination in the radiotherapy group and of 0.15 in the non-radiotherapy group, i.e. 1.3 times more often. Findings that turned out to be negative at confirmation were 2.0 times ($P < 0.05$) more common in the radiotherapy group. These false-positive findings were more common in the radiotherapy group than in the surgery group and only shortly after treatment. Mammography is more difficult to interpret after radiotherapy than after conservative surgery alone, especially shortly after treatment, and more often involves extra diagnostic tests and findings that will be negative at confirmation.

**Keywords:** breast cancer radiation, follow-up mammography

Because mammography has proved to be a valid test for screening it is assumed also to be effective in the follow-up of breast cancer patients. Operation and radiotherapy cause different changes in the breast that may cause difficulties in interpretation of mammography, resulting in underdiagnosis of recurrence. On the other hand, overdiagnosis in terms of a positive finding, which turns out to be negative at confirmation, causes unnecessary fear and uncertainty to the patient. The aim of this study was to ascertain whether follow-up mammography was more difficult to interpret after conservative treatment of breast carcinoma if radiotherapy was administered than if the breast was treated with segmental resection alone.

### PATIENTS AND METHODS

This prospective, randomized study was carried out in Tampere University Hospital during the years 1990–95. All consecutive low-risk breast cancer patients were randomized to have radiotherapy after segmental resection or to have no further therapy. Small invasive carcinomas ≤2 cm without evidence of an extensive intraductal component (EIC), node negative, histological grade I or II, c-*erb*B-negative, DNA-diploid with low S-phase fraction (≤ 8%), free margins in operation ≥ 1 cm and over 40-year-old woman were the criteria for low risk. Patients who had small tumour size (diameter ≤ 5 mm) were also considered to have low-risk breast cancer even if biological measurements were not performed. Hormone receptors were measured in 81% of cases and all were progesterone receptor (PgR)-positive. Ploidy and c-erbB were measured in 69% of cases and all of these were DNA diploid and c-erbB negative.

There were 144 patients included in the study during the 5-year accrual period. All patients had segmental resection along the fascia level with at least 1 cm free margins. The axilla was dissected via a separate incision to levels I and II. Patients were randomized after surgery to receive radiotherapy or not. Radiotherapy was commenced within 8 weeks after surgery. The whole breast and chest wall were included in the treated target volume. The total radiation dose was 50 Gy. Radiotherapy was given five times per week in 2 Gy per fraction. Radiotherapy was performed using the two opposing tangential fields technique by photons from a 2- to 6-MV linear accelerator. The isodose representing the minimum target-absorbed dose was defined as 95% of the specification dose. No boost or bolus was used. None of the patients received adjuvant hormone or chemotherapy.

The follow-up was organized in the oncological outpatient department. Clinical examination was performed every third month during the first year, every fourth month during the second and every sixth month thereafter. Follow-up mammography of both breasts was carried out routinely every 18 months and more frequently if clinically indicated. The first mammography was routinely carried out 18 months after the operation and earlier only if clinically indicated. Preoperative mammographs were available for all patients for comparison.

*Received 13 October 97*
*Revised 5 February 98*
*Accepted 9 February 98*

*Correspondence to:* K Holli

Mammograms were performed in the Department of Radiology of Tampere University Hospital, and two radiologists with long experience in mammography and working in the hospital interpreted the films in their routine clinical work blindly and even without knowing that the study was in progress.

The number of mammograms in the randomized groups was estimated per patient and per follow-up year. The number of extra diagnostic examinations (extra mammogram examinations, ultrasound, fine-needle aspiration or biopsy) and the occurrence of positive mammographic findings were assessed per mammography session and per follow-up year.

A routine mammography examination consisted of craniocaudal and mediolateral oblique views. After seeing these films the radiologist normally decides if a magnified view or ultrasound is necessary and these are taken during the same visit. This was not regarded as an extra examination. Only those cases that were regarded as unclear and in need of more clarification with other methods or repetition of the same kind of examinations as before were counted as positive findings and extra diagnostic examinations.

Two-sided *P*-values by Fisher's exact test were used to test the significance of the findings. Odds ratios and 95% confidence intervals are presented for the effect of time of follow-up to the extra diagnostic tests.

### RESULTS

There were 144 patients in all, 78 in the radiotherapy and 66 in the non-radiotherapy group. No differences were seen between the intervention groups in age of patients or other prognostic factors (Table 1). The characteristics of tumours and the surgical treatment were fixed in the inclusion criteria. The number of bilateral mammograms taken was 239 during the mean follow-up time of 2.9 years, i.e. 411 cumulative follow-up years (Table 2); this corresponded to 1.7 mammograms per patient and 0.6 mammograms per follow-up year. The number was about the same as planned, i.e. about one mammography examination every 18 months. There was no difference in this respect between the treatment groups.

Altogether 48 patients were sent to mammography earlier than originally scheduled (range 6–17 months) because of some suspicion in clinical examination or symptoms of the patient. However, for those who had normal findings the next mammography was delayed so that the average interval between exams was approximately as originally planned.

Further diagnostic tests after the routine mammography examination were more frequently called for in the radiotherapy group (27) than in the non-radiotherapy group (15) (Table 3). However, the difference was fairly small and not significant ($P = 0.14$). There were 0.19 further tests per mammography in the radiotherapy group and 0.15 in the non-radiotherapy group. The intensity per patient year was also about the same, 0.11 vs 0.09 in both groups (Table 3). In the radiotherapy group there were five biopsies, 14 fine-needle aspirations in ultrasound control and eight control mammography examinations with magnified view and ultrasound. In the non-radiotherapy group the numbers were three, six and six respectively.

The mammographic finding was classified as positive more frequently in the radiotherapy group (20) than in the non-radiotherapy (nine) group, but the difference was not significant ($P = 0.10$). Positive findings per mammography were 1.6 times more frequent in the group with radiotherapy (0.14) than in the group without (0.09). The positive finding was confirmed to be recurrent breast cancer, i.e. true positive, in three patients in each group, and two other patients in the radiotherapy group were operated on for fat necrosis. Thus, positive findings that turned out to be negative at confirmation were 2.0 times more frequent per mammography in the radiotherapy group (17 cases) than in the non-radiotherapy group (six cases), which was significant ($P = 0.04$) (Table 3).

Further tests were called for more often only at the beginning of follow-up (OR = 2.0 if follow-up < 18 and 1.0 if follow-up was ≥ 18 months). The tests were performed 4.3 times more often in the radiotherapy group than in the non-radiotherapy group (Table 4).

### DISCUSSION

The aim of population-based mammography screening is to reduce mortality from breast cancer by detecting preclinical disease. Mammography has high sensitivity and specificity in detecting preclinical breast cancer (Miller et al, 1991). This improvement in the diagnostic method will lead to a significant reduction in

**Table 1** The Tampere randomized trial on breast-conserving surgery with or without radiotherapy in breast cancer patients with good prognosis 1990–95. Patient characteristics by treatment

| Characteristic | Radiotherapy | | |
|---|---|---|---|
| | Yes | No | Total |
| Age (mean) | 55.6 | 56.3 | 55.9 |
| S-phase (mean) | 4.9 | 4.7 | 4.8 |
| Histology | | | |
| Ductal invasive | 69 (88%) | 57 (86%) | 126 (87%) |
| Lobular invasive | 7 (9%) | 7 (11%) | 14 (10%) |
| Other invasive | 2 (3%) | 2 (3%) | 4 (3%) |
| Grade | | | |
| I | 53 (68%) | 47 (71%) | 100 (70%) |
| Other | 25 (32%) | 19 (29%) | 44 (30%) |
| Total | 78 (100%) | 66 (100%) | 144 (100%) |

**Table 2** The Tampere randomized trial on breast-conserving surgery with radiotherapy (RT) vs without radiotherapy (No-RT) in breast cancer patients with good prognosis 1990–95. Number of patients, follow-up years and mammography examinations by treatment

| | RT | | No-RT | | Total | |
|---|---|---|---|---|---|---|
| | *n* | Per patient | *n* | Per patient | *n* | Per patient |
| Number of patients | 78 | | 66 | | 144 | |
| Follow-up years | 238 | 3.1 | 173 | 2.6 | 411 | 2.9 |
| Mammography | 142 | 1.8 | 97 | 1.5 | 239 | 1.7 |

Table 3 The Tampere randomized trial on breast-conserving surgery with radiotherapy (RT) vs without radiotherapy (No-RT) in breast cancer patients with good prognosis 1990–95. Number and frequency of true and false abnormal findings by treatment.

| Type of event | RT | | | No-RT | | | |
|---|---|---|---|---|---|---|---|
| | Number | Per mammography | Per follow-up year | Number | Per mammography | Per follow-up year | *P*-value |
| Further diagnostic test[a] | 27 | 0.19 | 0.11 | 15 | 0.15 | 0.09 | 0.14 |
| Positive finding | 20 | 0.14 | 0.08 | 9 | 0.09 | 0.05 | 0.10 |
| Recurrence | 3 | 0.02 | 0.01 | 3 | 0.03 | 0.02 | 0.19 |
| Uncertain findings | 17 | 0.12 | 0.07 | 6 | 0.06 | 0.03 | 0.04 |

[a]Further diagnostic tests: ultrasound, extra mammography, fine-needle aspiration and/or biopsy.

Table 4 Number, period prevalence (%) and odds ratio of further tests prompted by mammography by treatment and length of follow-up (months)

| Treatment | Interval from surgery to mammography examination | | | |
|---|---|---|---|---|
| | Less than 18 | | 18–60 | |
| | Number | Prevalence | Number | Prevalence |
| No radiation | 3/19 | 16 | 6/82 | 8 |
| Radiation | 12/44 | 27 | 8/108 | 8 |
| OR | | 2.0 | | 1.0 |

mortality from breast cancer for women of 50 years and older (Wald et al, 1991). Mammography screening also has other advantages besides possible reduction in mortality, i.e. early detection of breast cancer allows more conservative treatment and may have a positive effect on the quality of life of breast cancer patients (de Koning et al, 1992). Because mammography is a valid test for screening it is also assumed to be effective in the post-treatment follow-up of breast cancer patients (Sadowsky et al, 1990; Orel et al, 1992; Jager et al, 1995; American Society of Clinical Oncology, 1997).

The risk of a breast cancer patient developing a second cancer in the opposite breast is about five times as large as the risk of first breast cancer in the general population (Brenner et al, 1993). There is also the risk of a local relapse in the treated breast even if radiotherapy is given (Fisher et al, 1995). Radiotherapy is widely used because it prevents local relapses after conservative surgery (Fisher et al, 1995). The numbers of local relapses and second cancers in this study (three vs three) were too small for valid conclusions.

There is so far no evidence that routine follow-up for recurrence produces any useful diagnostic lead-time or prolongs survival (GIVIO Investigators, 1994). However, the routine use of mammography has generally been recommended for the contralateral and ipsilateral breast in women of any age with a history of breast cancer (American Society of Clinical Oncology, 1997). The recommendations are based on retrospective studies that have shown that mammography detects ipsilateral breast cancer recurrences and second cancers smaller than them are detected by physical examination (Mellik et al, 1991; Orel et al, 1992; Barr et al, 1993; Jager et al, 1995).

Radiotherapy changes the breast and causes fat necrosis, fibrosis, skin thickening and other parenchymal distortion, which might further cause diagnostic problems (Welch, 1986). In our randomized study the interpretation of mammography in normal clinical practice was more difficult after radiotherapy than after conservative surgery alone, and gave rise to extra diagnostic tests and positive findings. Patients have a fear of recurrence after breast cancer and diagnostic tests, and in particular a positive result may increase anxiety and fear (Marteau 1989, 1990). Waiting for the next mammography examination and knowing that the previous was not considered absolutely normal is stressful for the patient and should be avoided.

The false-positive mammography examination was more frequent at the beginning of follow-up, especially in the radiotherapy group. Extra tests were common in the radiotherapy group during the first 18 months but not after this as compared with patients treated with surgery alone. The optimal frequency of post-treatment mammography has not been established. It seems to vary from 6 to 24 months in clinical practice in different centres (Recht et al, 1989; Sadowsky et al, 1990; Orel et al, 1992; Rosselli del Turco et al, 1994; GIVIO Investigators, 1994). The American Society of Clinical Oncology has recently recommended that after conservative therapy the first mammography should be taken 6 months after completion of radiotherapy and annually after that. Our results indicate that local relapse is rare in low-risk patients and that mammograms shortly after radiotherapy cause extra tests and stress for the patient compared with mammograms after conservative surgery only. Furthermore, the breast is often swollen and painful after treatment and mammography may cause harm to the breast and inconvenience to the patient. We propose that the first post-treatment mammography should not be taken in the first $1^1/_2$ years after primary treatment, especially in patients with a low risk of recurrence and who have been subjected to radiation treatment.

### ACKNOWLEDGEMENTS

The authors are indebted to Mrs Marita Hallila for help. This study was financially supported by the Finnish Cancer Society and the Medical Research Fund of Tampere University Hospital.

© Cancer Research Campaign 1998

### REFERENCES

American Society of Clinical Oncology (1997) Recommended breast cancer surveillance guidelines. *J Clin Oncol* **15**: 2149–2156

Barr LC, Skene AI, Fish S and Thomas JM (1993) Post-treatment mammography following the breast-conserving treatment of breast cancer: is it of value. *Breast* **2**: 253–254

Brenner H, Engelsmann B, Stegmaier C and Ziegler H (1993) Clinical epidemiology of bilateral breast cancer. *Cancer* **72**: 3629–3635

de Koning HJ, van Ineveld BM, de Haes JCJM, van Oortmarssen GJ, Klijn Jr GM and van den Maas PJ (1992) Advanced breast cancer and its prevention by screening. *Br J Cancer* **65**: 950–955

Fisher B, Anderson S, Redmond CK, Wolmark N, Wickerham L and Cronin WM (1995) Reanalysis and results after 12 years of follow-up in a randomized clinical trial comparing total mastectomy with lumpectomy with or without irradiation in the treatment of breast cancer. *N Engl J Med* **333**: 1456–1461

GIVIO Investigators (1994) Impact of follow-up testing on survival and health-related quality of life in breast cancer patients; a multicentre randomized controlled trial. *JAMA* **271**: 1587–1592

Jager JJ, Langendijk JA, Dohmen JP, Schreutelkamp JL, Volovics L, van Engelshoven JM, de Jong JM, Schouten LJ, Hupperets PS and Blijham GH (1995) Mammography in the follow-up after breast-conserving treatment in cancer of the breast: suitability for mammographic interpretation, validity and interobserver variation. *Br J Radiol* **68**: 754–760

Marteau T (1989) Psychological costs of screening. *Br Med J* **299**: 527

Marteau T (1990) Reducing the psychological costs. *Br Med J* **301**: 26–28

Mellink WAM, Holland R, Hendrichs JHCL, Peeters PHM, Rutgers EJT and van Daal WKJ (1991) The contributions of routine follow-up mammography to an early detection of asynchronous contralateral breast cancer. *Cancer* **67**: 1844–1848

Miller AB, Chamberlain J, Day NE, Hakama M, Prorok PC (eds) (1991) *Cancer Screening. UICC Project on Evaluation of Screening for Cancer.* International Union Against Cancer: Cambridge

Orel SG, Troupin RH, Patterson EA and Fowble BL (1992) Breast recurrence after lumpectomy and irradiation: role of mammography in detection. *Radiology* **183**: 210–216

Recht A, Schnitt SJ, Connolly JL, Rose MA, Silver B, Come S, Henderson C, Slavin S and Harris JR (1989) Prognosis following local or regional recurrence after conservative surgery and radiotherapy for early stage breast carcinoma. *Int J Radiat Oncol Biol Phys* **16**: 3–9

Rosselli del Turco M, Palli D, Gariddi A, Giatto S, Pacini P and Distante V (1994) Intensive diagnostic follow-up after treatment of primary breast cancer. *JAMA* **271**: 1593–1597

Sadowsky NL, Semin A and Harris JR (1990) Breast imaging: a critical aspect of breast conserving treatment. *Cancer* **65**(9 Suppl.): 2113–2118

Wald N, Frost C and Cuckle H (1991) Breast cancer screening; the current position. *Br Med J* **302**: 845–846

Welch JS (1986) The post-irradiated breast. *Mayo Clin Proc* **61**: 392–395

© Cancer Research Campaign 1998